# Criterion-Related Validity of the Sugar-Sweetened Beverage (SSB) Questionnaire Among Children and Adolescents in Malaysia

**DOI:** 10.7759/cureus.61984

**Published:** 2024-06-09

**Authors:** Nur Arina Bakeri, Nur Amalina Amirullah, Norhasmah Sulaiman, Wan Ying Gan, Su Peng Loh, Salma Faeza Ahmad Fuzi, Siti Raihanah Shafie, Nazli Suhardi Ibrahim, Fatimah Zurina Mohamad, Rusidah Selamat

**Affiliations:** 1 Department of Nutrition, Universiti Putra Malaysia, Serdang, MYS; 2 Nutrition Division, Ministry of Health Malaysia, Putrajaya, MYS

**Keywords:** sugar-sweetened beverages, overweight, obesity, criterion-related validity, children, adolescents

## Abstract

Sugar-sweetened beverages (SSBs) are a major source of dietary sugar, and their consumption is on the rise among children and adolescents. Excessive sugar intake is a significant contributor to overweight, obesity, and non-communicable diseases (NCDs). The consumption of SSBs, particularly that of children and adolescents, has been of interest as of late, as they are implicated in affecting body weight status. Thus, the goal of this study was to determine the predictive criterion-related validity of the SSB questionnaire that was administered to children and adolescents to assess their SSB and non-SSB intake. A nationwide cross-sectional study involving 5211 respondents aged 7-17 years old and their parents was conducted. The self-administered Malay questionnaire was distributed to collect information on socioeconomic background, the frequency of eating out at restaurants or other food premises, the availability of SSBs at home, and SSB consumption patterns of children and adolescents within a week. The predictive criterion-related validity was determined by using six hypotheses that can differentiate between two independent sample means of SSB consumption based on age, gender, locality, monthly household income, frequency of eating out at restaurants or other food premises, and availability of SSBs at home. The independent samples t-test and one-way ANOVA were used to conduct the validation process. Five out of six hypotheses were accepted*. *Significant mean differences were observed between sociodemographic factors, such as age (*t*=-10.56, *p*<0.001), localities (*t*=-5.37, *p*<0.001), monthly household income (*F*=26.83, *p*<0.001), and SSB consumption. Behavioural factors, including eating out at restaurants or other food premises (*t*=9.93, *p*<0.001) and environmental factors such as the availability of SSBs at home (*F*=136.24, *p*<0.001) also showed a significant difference with SSB consumption. The SSB questionnaire demonstrated the ability to differentiate between groups. Thus, this SSB questionnaire appears to be valid to measure the SSB consumption of children and adolescents.

## Introduction

Sugar-sweetened beverages (SSBs) constitute a primary source of dietary sugar, and their consumption is increasing among children and adolescents [1]. In these few years, the term SSBs has now begun to be used to represent any beverages containing added sugar. Until today, there were inconsistencies and no standardised definitions used for categorising SSBs. According to the World Health Organization (WHO) [1], SSBs are defined as "all beverages containing free sugars, i.e., carbonated or non-carbonated soft drinks, 100% fruit/vegetable juices and drinks, liquid and powder concentrates, flavoured water, energy and sports drinks, ready-to-drink tea, ready-to-drink coffee, and flavoured milk drinks." A review by Malik and Hu defined SSBs as "beverages that contain added sugar, including carbonated and non-carbonated soft drinks, fruit drinks, and sports and energy drinks that are typically low in nutritional quality" [2]. Instant coffee or tea is commonly included in the SSB category in some local studies in Malaysia [3,4]. A plausible reason for the unstandardised SSB definition in each study from different countries might be due to the culture of the places of interest: instant drinks might not be typically consumed in certain places, while milk might not be consumed on a daily basis in other places. Discrepancies in SSB categorisation led to some challenges when comparing the volume of SSB consumed.

A study by McCormick et al. [5] reported that children aged 11-13 years old in the United States consumed 1073.5 mL/day of SSBs, while another study in Mexico reported that children aged 12-19 years old consumed about 231.2 mL/day of SSBs [6]. Meanwhile, in Malaysia, the daily SSB consumption among school-aged children between 10 and 17 years old was reported to be 345.1 mL/day [4]. In another local study, it was reported that the SBB consumed by children aged between 12 and 16 years old was 1038.2 mL/day [3]. The huge differences in SSB consumption among these studies might be due to the different techniques of data collection used, such as self-administered questionnaires vs. interviews. Higher SSB consumption was observed among those who self-reported their SSB consumption, while studies that used the interview method yielded a lower volume of SSB consumption. This showed that self-reported data tend to overestimate the volume of SSB consumed.

Several studies proved that higher SSB consumption was highly related to the increasing trend of childhood overweight and obesity [7]. The escalating interest in assessing SSB consumption has resulted in the need to develop a valid, reliable, and standardised SSB consumption questionnaire. A standardised SSB consumption questionnaire is progressively recommended as a suitable SSB consumption measure in any population survey. Nevertheless, before this questionnaire is to be adopted as a universal tool for SSB consumption measurement, it is imperative to ascertain its operating characteristics.

With the growing concerns related to SSB consumption among children, various types of questionnaires have been used to measure SSB consumption in previous studies. The most common questionnaires used are the Food Frequency Questionnaire (FFQ) [3,4,6,8-10] and the Beverage Intake Questionnaire (BEVQ-15) [5,11]. Meanwhile, some other studies used dichotomous responses, such as whether the respondents consumed SSBs in the past week or not [12,13]. Different questionnaires used to measure SSB consumption have led to some challenges in measuring and comparing the consumption status of different populations. One of the challenges includes measuring the volume of SSB consumed per day according to its categories. Even within similar populations (i.e., Malaysian adolescents), the use of different questionnaires could potentially yield different results.

The 24-hour dietary recall is known to be the most accurate in assessing food and nutrient intake at present [14]. It provides detailed information on food or beverages consumed over the past 24 hours. Nevertheless, the 24-hour dietary recall requires a trained interviewer, is expensive, and is time-consuming, as multiple days of assessments are needed to assess the usual intake of the respondents [15]. Besides, the FFQ has been widely used in large epidemiological studies since the 1990s due to its cost-effectiveness, time-saving capacity, and capability to evaluate an individual's typical dietary intake [14,15]. However, FFQ is exposed to recall bias as the respondent needs to recall the food or drink consumed over the past month and year and the long lists of food and beverages in FFQ burden the respondents. Thus, this SSB consumption questionnaire was developed to assess the intake of the respondents in a particular week without overburdening them and to reduce the time consumed to answer the questionnaire. 

Given the uncertain universality of any measurement tool, it is important to evaluate a questionnaire's suitability for diverse purposes and various demographic groups, especially in Malaysia's context. Predictive criterion-related validity refers to the extent to which a measure is related to an outcome and its ability to differentiate between groups of people and predict future outcomes [16]. In this paper, a cross-sectional study was conducted to assess the SSB consumption of children and adolescents in Malaysia and how well this questionnaire was in distinguishing SSB consumption between groups such as age, gender, locality, monthly household income, eating out at restaurants or other food premises, and availability of SSBs at home.

## Materials and methods

Location and study population

This cross-sectional study spanned across all 16 states and federal territories in Malaysia and involved 123 public primary and secondary schools. The study focused on children (7-9 years old) and adolescents (10-17 years old) in Malaysia, who were chosen based on specific inclusion and exclusion criteria. This study excluded any children or adolescents who self-reported their medical conditions (e.g., hypertension, diabetes, cardiovascular disease, thyroid disease, asthma, food allergies, eczema), Standard 6 students, and those who were taking the Form Three Assessment (PT3) or Malaysian Certificate of Education (SPM) examination. Children and adolescents from boarding schools, private schools, international schools, and religious schools were also excluded from the study. This study was conducted from January 17 until August 26, 2022.

Ethical approval

Ethical approval was obtained from the Ethics Committee for Research Involving Human Subjects at Universiti Putra Malaysia (reference number: JKEUPM-2022-029). The participants and their parents were provided with a detailed overview of the study and were asked to sign informed consent forms prior to their involvement in the study.

Sampling frame

The survey utilised the Ministry of Education's list of primary and secondary schools as the sampling frame. The minimum sample size needed for the analysis was determined using a formula for a prevalence study with a 95% confidence interval and a 5% margin of error. p is the expected prevalence of carbonated drink consumption at least once a day in the past 30 days based on data from the National Health and Morbidity Survey (NHMS) 2017 (p=0.369) [17]. The resulting sample size was calculated at 358. For an optimum sample size, adjustments were made to account for a design effect of 2, a response rate of 80%, and specific analysis requirements, such as comparisons between rural and urban areas, boys and girls, and age groups of 7-9 years old versus 10-17 years old. Consequently, the final required sample size was determined to be 7160 respondents.

The study employed multistage stratified cluster sampling and a systematic random approach to choose a minimum of one to a maximum of 21 classes from each selected school. Every student in the selected classes was invited to participate in the study.

Instruments

The study used two distinct sets of self-administered questionnaires for children (aged 7-9 years old) and adolescents (aged 10-17 years old) in Malay language. The questionnaires for the children were completed by their parents or caregivers (herewith referred to only as parents), who are responsible for household food and beverage purchases. On the other hand, the questionnaires for the adolescents were filled out by the adolescents themselves and their parents in the respective sections. In cases where the respondents had a disability or impairment affecting their ability to read or make judgements (e.g., visual, physical, learning, or mental disability), the parents were responsible for answering the questionnaires on their behalf.

The factors that were investigated include age, gender, localities, monthly household income, eating out practices such as eating out at restaurants or other food premises [17,18], and the availability of SSBs at home [19]. Gross monthly income classifications (B40, M40, and T20) were based on the Household Income and Basic Amenities Survey (HIS) Report 2019 [20]. Monthly income classifications were as follows: low income, B40 (≤MYR 4849 or ≤USD 1042); middle income, M40 (MYR 4850-10959 or USD 1043-2356); and high income, T20 (≥MYR 10960 or ≥USD 2357).

The SSB consumption questionnaire was adapted from the Beverage Intake Questionnaire (BEV-Q) [21] with several modifications based on NHMS 2014 [22], the Ministry of Health Malaysia, the Food Regulation 1985 [23], and the WHO [1]. The list of SSBs included in the questionnaire were any ready-to-drink beverages such as (1) carbonated drinks, (2) isotonic drinks, (3) energy drinks, (4) sweetened coffee or tea, (5) botanical beverages, (6) malted drinks, (7) flavoured and (8) unflavoured full cream milk, (9) 100% fruit juice, (10) sweetened juice beverages, (11) vegetable juice, and (12) cultured milk drinks, especially the commercially produced and pre-packed beverages. Other beverages that were not categorised as SSBs included (13) plain water, (14) alcoholic beverages, (15) diet carbonated drinks, (16) premix drinks, (17) boba milk tea, (18) specialty drinks, and (19) others. The SSB consumption of children and adolescents was assessed by the type of SSB consumed, the frequency (times/week), and the serving size (mL/day). The total volume of beverages consumed per day (mL/day) was calculated using the following formula: total volume of beverage consumed per day (mL/day)=frequency of beverage consumed per week (times)×total volume of beverage consumed per serving (mL)/7.

The total volume of SSB consumed was summed up within the SSB categories. The limit for total SSB consumption was set at 4000 mL/day. The cut-off was set according to previous studies [24,25], which aimed to reduce the potential risk of over-reporting beverage intake among respondents.

Data analyses

The collected data were analysed using IBM SPSS Statistics for Windows, Version 26.0 (Released 2019; IBM Corp., Armonk, New York, United States). Continuous variables were assessed for normality by examining the skewness of the data within the range of ±2 [26]. Categorical data were presented in frequencies and percentages, while continuous data were reported in means and standard deviations or medians and interquartile ranges (IQR) for data that were not normally distributed. Due to the limited references on cut-off points in selected variables, such as the availability of SSBs at home, the tertile method was used to classify these variables into groups. The predictive criterion-related validity of the SSB consumption questionnaire was measured by bivariate analyses, including an independent samples t-test and a one-way ANOVA. Both analyses were conducted to compare the mean SSB consumption according to age, gender, locality, monthly household income, eating out practices at restaurants or other food premises, and the availability of SSBs at home. A post hoc analysis (Bonferroni test) was conducted to determine any significant differences in the mean SSB consumption between groups. The significance level was set at p<0.05.

Six hypotheses were identified to determine the predictive criterion-related validity, which are the following: (1) the volume of SSB consumption would be significantly higher among adolescents than children, (2) the volume of SSB consumption would be significantly higher among female children and adolescents than male children and adolescents, (3) the volume of SSB consumption would be significantly higher among those who lived in rural areas compared to urban areas, (4) the volume of SSB consumption would be significantly higher among the B40 (low) income group compared to the M40 (middle) and T20 (high) income groups, (5) the volume of SSB consumption would be significantly higher among those who ate out at restaurants/ate out within a week compared to those who did not, and (6) the volume of SSB consumption would be significantly higher among those who had high availability of SSBs at home compared to those who had low or moderate availability of SSBs at home.

Since the independent variables were categorical and the dependent variable was continuous, an independent samples t-test and a one-way ANOVA were used.

## Results

Descriptive analysis

Out of 7160 questionnaires distributed to respondents aged 7-17 years old, 5491 were collected, with a response rate of 76.7%. After the data cleaning process, only 5211 respondents' data were used in this study. Table 1 summarises the sociodemographic characteristics, behavioural factors, and environmental factors of the respondents. The mean age of the respondents was 12.01±3.13 years old. There were about 2247 males (43.1%) and 2962 females (56.9%) involved in this study. Half of the respondents lived in urban areas (49.6%, n=2587). The mean number of household members was 5.6±1.81. Most of the parents were married (91.7%, n=4581), and the highest education levels for both parents were at the secondary level (54.2%, n=2598 for the mother and 55.3%, n=2460 for the father). The median (IQR) total monthly household income was MYR 2000 (MYR 5000-1000) or USD 427 (USD 1067.50-213.50); meanwhile, the median (IQR) monthly household SSB expenditure was MYR 50 (MYR 100-25) or USD 10.70 (USD 21.40-5.30). In terms of household income levels, 3571 respondents were categorised as B40 (74.1%), 926 respondents were M40 (19.4%), and only 285 respondents were T20 (6.0%). 

More than half of the respondents consumed SSBs while eating at restaurants or other food premises (63.4%, n=3304). This investigation discovered that 22.2% (n=1131) of the respondents fell into the first tertile for the availability of SSB at home, 44.6% (n=2276) in the second tertile, and 33.3% (n=1698) in the third tertile (Table 1).

**Table 1:** Sociodemographic characteristics and behavioural and environmental factors of the respondents (n=5211). ^1^Gross monthly income classifications (B40, M40, T20) are based on the Household Income and Basic Amenities Survey (HIS) Report, 2019, the Department of Statistics, Malaysia. Monthly income classifications were as follows: B40 (≤MYR 4849 or ≤USD 1042); M40 (MYR 4850-10959 or USD 1043-2356); and T20 (≥MYR 10960 or ≥USD 2357). The data has been represented as n, %, mean±SD, and median (IQR). SSBs: sugar-sweetened beverages

Variable	Category	n	%	Mean/median	SD/IQR
Sociodemographic factors					
Age category (years)	Children (7-9 years old)	1667	32.1	12.01	3.13
	Adolescent (10-17 years old)	3525	67.9		
Gender	Male	2247	43.1	NA/-	NA/-
	Female	2962	56.9		
Localities	Urban	2587	49.6	NA/-	NA/-
	Rural	2624	50.4		
Ethnicity	Bumiputera	4646	90	NA/-	NA/-
	Non-bumiputera	517	10		
Number of family members	Less than 5 members	1232	25.9	5.60	1.81
	5 members and more	3519	74.1		
Parental marital status	Married	4581	91.7	NA/-	NA/-
	Divorced/separated/widow/widower	412	8.3		
Mother's education level	Never attended school	73	1.5	NA/-	NA/-
	Primary	450	9.4		
	Secondary	2598	54.2		
	Tertiary	1649	34.4		
	Others	25	0.5		
Father's education level	Never attended school	58	1.3	NA/-	NA/-
	Primary	507	11.4		
	Secondary	2460	55.3		
	Tertiary	1407	31.6		
	Others	19	0.4		
Disability impairment	Yes	5030	97.2	NA/-	NA/-
	No	147	2.8		
Monthly household income (MYR/month)^1^ (median)	B40 (≤MYR 4849)	3571	74.7	2000	5000.00-1000.00
	M40 (MYR 4850-10959)	926	19.4		
	T20 (≥MYR 10960)	285	6		
Weekly pocket money (MYR/week) (median)	Less than MYR 10/week	1435	28.8	15	20.00-5.00
	MYR 10-19/week	1860	37.3		
	MYR 20/week and more	1695	34		
Monthly household SSB expenditure (MYR/month) (median)	Less than MYR 40/month	1485	33.6	50	100.00-25.00
	MYR 40-99/month	1304	29.5		
	MYR 100/month and more	1632	36.9		
Behavioural factor					
Eating from restaurants/other premises in a week (days/week)	Yes	3304	63.4	1.19	1.34
	No	1907	36.6		
Environmental factor					
Availability of SSBs at home	1st tertile (below 5)	1131	22.2	6.64	2.90
	2nd tertile (5-7)	2276	44.6		
	3rd tertile (8 and above)	1698	33.3		

SSB consumption among children and adolescents

Table 2 shows the consumption of SSB among the respondents. The mean total volume of SSB consumption of the respondents was 758.79±733.15 mL/day, which was equivalent to approximately 3 glasses/day or 2.3 cans of SSBs/day.

**Table 2:** SSB consumption of children and adolescents. The data has been represented as n, %, and mean±SD. SSB: sugar-sweetened beverage

Variable	Category	n	%	Mean	SD
SSB consumption of children and adolescents (7-17 years old)	1st tertile (<319mL/day)	1558	33.2	758.8	733.15
	2nd tertile (320-790mL/day)	1563	33.4		
	3rd tertile (≥791mL/day)	1563	33.4		

Predictive criterion-related validity

Table 3 shows the results of an independent samples t-test and a one-way ANOVA to determine the predictive criterion-related validity. All hypotheses, except the second hypothesis, reported high validity in distinguishing the means of the groups. The mean SSB consumption was significantly different among children and adolescents, with adolescents consuming higher SSBs (833.9±776.5 mL/day) compared to children (593.6±599.0/day; t=-10.56, p<0.05). Females were revealed to consume slightly higher SSBs (766.9±733.7 mL/day) compared to male respondents (748.5±732.5 mL/day), but the differences were not statistically significant (t=-0.85, p>0.05). Besides, urban respondents were discovered to consume fewer SSBs (699.7±708.4 mL/day) compared to their counterparts in rural areas (814.4±751.6 mL/day; t=-5.37, p<0.05). Moreover, the findings showed that higher monthly household income was related to lower SSB consumption (F=26.83, p<0.05). The post hoc analysis revealed that the B40 group had significantly higher SSB consumption (810.3±755.6 mL/day) than the M40 group (633.7±643.7 mL/day) and the T20 group (602.5±602.8 mL/day). However, there was no significant difference in terms of SSB consumption between the M40 group and the high T20 group. This study also revealed that those who frequently ate outside at restaurants or other food premises consumed higher SSBs compared to those who did not (t=9.93, p<0.05). In addition, environmental factors also played a major role in influencing children's and adolescents' consumption of SSBs. Based on the findings, the mean SSB consumption of the respondents was significantly higher with the availability of SSBs at home (F=136.24, p<0.05).

**Table 3:** Differences in SSB consumption among children and adolescents based on the factors. The data has been represented as n, mean±SD, F or t, and p-value. *p<0.05 ^a^Independent samples t-test ^b^One-way ANOVA ^c^Significant difference between groups 1 and 2 ^d^Significant difference between groups 1 and 3 ^e^Significant difference between groups 2 and 3 SSB: sugar-sweetened beverage

Variable	Category	n		SSB consumption (mL/day)		F or t	P-value		Interpretation
				Mean	SD				
Sociodemographic factors									
Age category^a^	Children	1491		593.6	599	-10.56	<0.05*		Accept hypothesis
	Adolescent	3179		833.9	776.5				
Gender^a^	Male	2018		748.5	732.5	-0.85	0.395		Reject hypothesis
	Female	2668		766.9	733.7				
Localities^a^	Urban	2273		699.7	708.4	-5.37	<0.05*		Accept hypothesis
	Rural	2414		814.4	751.6				
Monthly household income^b^	B40	3226		810.3^c,d^	755.6	26.83	<0.05*		Accept hypothesis
	M40	849		633.7^c^	643.7				
	T20	268		602.5^d^	602.8				
Behavioural factors									
Eating from restaurants/other premises in a week^a^	Yes		2970	838.8	768.5	9.93		<0.05*	Accept hypothesis
	No		1717	620.3	644.7				
Environmental factors									
Availability of SSBs at home^b^	1st tertile (below 5)		1007	506.8^c,d^	603	136.24		<0.05*	Accept hypothesis
	2nd tertile (5-7)		2093	721.8^c,e^	694.1				
	3rd tertile (8 and above)		1529	974.9^d,e^	797.7				

## Discussion

To validate the SSB consumption questionnaire, a predictive criterion-related validity method was used to assess its ability to distinguish between groups, which it should theoretically be able to do [16]. We compared six samples for the validation process: children and adolescents, male and female, urban and rural, B40, M40, and T20, eating out at restaurants within a week, those who did not, and those who had low, moderate, or high availability of SSBs at home. These six factors were chosen based on strong evidence from previous studies showing that there were significant differences between the groups and a significant contribution towards the children's SSB consumption.

The current SSB questionnaire was seen to yield the intended results, such as assessing the volume of SSB consumption among the respondents. A previous review article mentioned that the median number of food and beverage lists included in 227 studies that used FFQ as an instrument was 79, with a range of 5-350 [27]. There are 19 items from different types of beverages listed in this SSB questionnaire (including 12 items of SSBs), which was considered to be the optimal number for assessing SSB consumption without overburdening the respondents. From this study, the reported mean volume of daily SSB consumption among the respondents aged 7-17 years old (758.8 mL/day) was quite high compared to the national study in 2017 (345.1 mL/day), which involved respondents from 10 to 17 years old only [17]. Meanwhile, another local study by Gan et al. reported that the consumption of SSBs among respondents aged 12-16 years old was 1038.15 mL/day [3]. The possible reason behind the differences might be due to the age differences and categories of SSBs in each study. Besides, since the study by Gan et al. [3] used self-administered questionnaires while the study by the Institute of Public Health [17] used interviews, the different techniques of data collection between both local studies could also potentially result in huge differences in the volume of SSB consumption.

The results exhibited that adolescents consumed higher SSBs compared to children, and these findings were supported by a study in China involving children aged 6-17 years old [9]. Other studies justified that older children would have more power and freedom over their food choices, especially when they were given pocket money [17]. This explanation is consistent with this study, where higher weekly pocket money of the children and adolescents was related to higher SSB consumption.

Besides, there were no significant differences between the SSB consumption of male and female respondents in this study. These findings are contradictory to some previous studies. The non-significant differences in SSB consumption could potentially be due to the different categorisations used for SSBs in different studies. For example, one study in Ottawa, Canada, found that females consumed higher SSBs. But the SSBs defined in the study only included carbonated drinks, sports drinks, and pre-sweetened tea or coffee, while 100% fruit juice was excluded [28]. The authors also did not mention whether milk (both flavoured and unflavoured) was included. This was in contrast with our study, where there were additional categories for SSBs, such as fruit juices, flavoured milk, and yoghurt drinks. Therefore, when more types of beverages were investigated, fewer differences were detected in SSBs consumed by both genders. This could explain why our study did not find significant differences between genders and SSB consumption.

This study also found that children and adolescents in rural areas consumed higher SSBs than their urban counterparts. These results were supported by a local study, which found rural children consumed higher SSBs than urban children [4], as well as by a national study [17], which discovered that adolescents in rural areas consumed more carbonated drinks than adolescents in urban areas. A qualitative study in rural southwestern United States mentioned that people who lacked access to clean and safe drinking water opted to consume SSB instead [29]. Another reason for the low consumption of SSBs among urban respondents might be due to the abundance of awareness programs being held at schools in urban areas compared to rural areas. A previous study in rural Appalachia of the United States also revealed that adolescents in rural areas consumed three times more SSBs than the national average [5].

Besides that, this study discovered that monthly household income influenced the SSB consumption of children and adolescents, with those from the B40 group consuming higher SSBs compared to their counterparts from the M40 and T20 groups. Several other studies also reported similar observations, where children from lower-income households were more likely to consume SSBs compared to children from higher-income households [9].

Other than that, children and adolescents who frequently ate out tended to consume higher SSBs compared to their counterparts. A previous study also concluded that SSB consumption increased with the increased frequency of eating out among children and adolescents [3]. This is because food outlets offer a variety of SSBs to choose from compared to home, as the availability of SSBs at home relies solely on the purchases made by the parents or household members. This highlights the importance of applying environmental cues at food outlets, such as health-related posters and traffic light labels on beverage menus and packaging, which could facilitate children's healthier food choices while eating out [30]. The findings were supported by a previous study, where a high density of stores around home and school increased children's accessibility to SSBs, thus influencing their SSB consumption [6].

This study found that the high availability of SSB at home was associated with high SSB consumption. These findings were consistent with other studies [3]. The association indicated that higher availability of SSBs increased children's accessibility to SSBs at home. This highlights parental responsibility in ensuring the availability and consumption of healthy beverages, including plain water, in the family.

There were also several limitations in this study. Firstly, the data collection was conducted during the COVID-19 pandemic, specifically during the fourth phase of the National Recovery Plan (NRP), which started on October 3, 2021. This situation caused limited movement and availability of respondents during the data collection process. To note, schools were allowed to reopen in stages during this phase, which permitted students to physically attend schools in a rotation system. This was done to avoid mass gathering and to enforce physical distancing between students throughout the learning session. Once the schools had achieved a certain percentage of vaccinated students, the students were allowed to attend school as usual without any rotation or home-based learning sessions. There was no specific date to begin a normal school session, as there were differences between schools and between states. Due to these conditions, the questionnaires used in this study had to be self-administered, as the researchers were restricted from directly meeting respondents. Besides, the Movement Control Order (MCO) conducted by the Malaysian government affected the accessibility of the respondents towards SSBs and their habitual meal patterns after the pandemic struck. Secondly, this study only focused on the predictive criterion-related validity process. Further validation processes that used the gold standard, such as 24-hour diet recall, are still in progress and will be discussed in future studies. Finally, the questionnaires used in this study were self-reported, which could result in under-reporting or over-reporting of the data.

Regardless of these limitations, this study could facilitate effective and efficient data collection on SSB consumption. There are several features of the questionnaire shown to be helpful to the respondents, such as detailed instructions to guide respondents and the addition of visual aids, such as household measurement tools or commercial packaging size. These helped the respondents visualise the actual size or portion of the beverage. This could potentially minimise the underestimation or overestimation of the beverages' portion sizes. Besides, this SSB questionnaire consists of 19 items (including 12 SSB items), which were seen to be an optimal number of items to assess the SSB consumption of the respondents. This could help reduce the time spent by the respondents answering the questionnaire, lessen their burden, reduce the under- or overestimation of SSB consumption reported by them, and lower the labour cost spent on the data collection process.

## Conclusions

The questionnaire used in this study was discovered to be a valid measure of individuals' beverage consumption, particularly in terms of group comparisons between age, gender, locality, monthly household income, eating out at restaurants or other food premises, and the availability of SSBs at home. The findings of this study showed that the SSB consumption of children and adolescents was influenced by factors such as sociodemographic factors, including children's age, locality, and monthly household income; behavioural factors, including eating out habits; and environmental factors, including the availability of SSBs at home. The comparison was mainly to assess the accuracy of this questionnaire to measure the volume of beverage consumed by individuals and the questionnaire's ability to distinguish SSB consumption between the groups. This study demonstrated that the SSB questionnaire was explicit, consistent with previous literature, and could yield the intended results when compared with results from other studies. Further validation of this SSB questionnaire is currently under way, where it compares this questionnaire with the gold standard, such as the 24-hour diet recall. Questionnaire validation in various groups, such as adults and athletes, should be considered for future studies too. Besides, effective intervention programs, according to the factors discovered in this study, should be conducted and expanded among the targeted groups. Policies related to the environment and media should be reinforced and strengthened to improve public health. Adopting this questionnaire in future studies could improve the data collection process and analysis of beverage consumption among targeted populations, especially SSBs.
